# A Novel Mouse Model of Traumatic Optic Neuropathy Using External Ultrasound Energy to Achieve Focal, Indirect Optic Nerve Injury

**DOI:** 10.1038/s41598-017-12225-6

**Published:** 2017-09-18

**Authors:** Wensi Tao, Galina Dvoriantchikova, Brian C. Tse, Steven Pappas, Tsung-Han Chou, Manuel Tapia, Vittorio Porciatti, Dmitry Ivanov, David T. Tse, Daniel Pelaez

**Affiliations:** 1 Dr. Nasser Al-Rashid Orbital Vision Research Center, Bascom Palmer Eye Institute, Department of Ophthalmology; University of Miami Miller School of Medicine, Miami, FL 33136 USA; 2 Department of Microbiology and Immunology; University of Miami Miller School of Medicine, Miami, FL 33136 USA; 3 Department of Biomedical Engineering, University of Miami College of Engineering, Coral Gables, FL 33146 USA

## Abstract

Traumatic optic neuropathy (TON) is a devastating cause of permanent visual loss following blunt injury to the head. Animal models for TON exist, but most fail to recapitulate the clinical scenario of closed head indirect trauma to the nerve and subsequent neurodegeneration. Thus, we developed a clinically-relevant animal model for TON using a novel ultrasonic pulse injury modality (sonication-induced TON; SI-TON). To trigger TON, a microtip probe sonifier was placed on the supraorbital ridge directly above the entrance of the optic nerve into the bony canal. An ultrasonic pulse was then delivered to the optic nerve. After injury, the number of RGCs in the retina as well as visual function measured by PERG steadily decreased over a two-week period. In the optic nerve, pro-inflammatory markers were upregulated within 6 hours following injury. Immunohistochemistry showed activation of microglia and infiltration of CD45-positive leukocytes in the optic nerve and initiation of a gliotic response. The SI-TON model is capable of delivering a non-contact concussive injury to the optic nerve and induce TON in mice. Thus, our data indicate that the SI-TON model reliably recapitulates the pathophysiology and progressive neurodegeneration seen in the human manifestation.

## Introduction

Traumatic optic neuropathy (TON) is an uncommon but devastating cause of permanent visual loss following blunt force trauma to the orbit. The incidence of TON ranges from 0.5–5% of all closed head injuries, and 2.5% of maxillofacial trauma and mid-face fractures^1^. Clinically, a patient with acute TON may present with either partial or complete loss of vision, an afferent pupillary defect, normal appearing optic disc or visual field defects^2^. With time, optic disc pallor will manifest. Histopathologically, TON results in axonal degeneration and retinal ganglion cell (RGC) death.^3^ Although TON has been the subject of substantial basic and clinical research, insights into its basic pathophysiologic and molecular mechanisms remain limited^2^.

TON is classified into two categories: direct and indirect^4^. Direct injury occurs when a foreign body or bone fragment causes damage by coming into direct contact with the optic nerve. More commonly, indirect optic nerve injury occurs when deformational forces are transferred through the bones of the skull or by globe torsion against the optic nerve^3,5^. Putative mechanisms of indirect injury include: transmission of concussive shock waves propagating through the orbit against the intra-orbital segment of the nerve^2^; momentum of the globe and orbital contents being absorbed by the fixed portion of the optic nerve at the entrance to the optic canal^3^; and compression of the intracanalicular segment of the optic nerve by the malleable bones of the optic canal^6^. The initial traumatic event initiates a cascading sequence of metabolic events that are believed to exacerbate the optic nerve injury^5^.

Currently, no evidence-based therapy exists to effectively treat TON^3,7^. In a prospective, comparative, non-randomized interventional study of TON visual outcomes treated with corticosteroids, optic canal decompression or observation, the International Optic Nerve Trauma Study assessed 133 patients with indirect TON treated within 7 days of injury^8–10^_._ After adjusting for baseline visual acuity, no clear benefit of either corticosteroids therapy or optic canal decompression surgery was observed^11^. The conclusion of the study was neither corticosteroids nor optic canal decompression should be considered the standard of care in patients with TON^12,13^. Thus, TON is an orbital condition in which effective therapy for vision recovery remains elusive.

Several animal models have been described to study the molecular and cellular underpinnings of TON injury. The three common models to study optic nerve trauma are optic nerve crush (ONC)^14^, axotomy^15^, and ocular blast^16^. The optic nerve crush (ONC) model is widely used wherein the optic nerve is surgically exposed and clamped with forceps or a hemostat for varying amounts of time^17^. The crush model causes local destruction of tissue ultrastructure and alteration of vascular plexus surrounding the globe and nerve^18^. Moreover, the model does not quantify the force applied to achieve injury^19^, and the degree of injuries are variable depending on the individual performing the procedure. In addition to severing the blood supply to the posterior segment of the globe^14^, the optic nerve transection^14^ technique shares many of the drawbacks of the crush model in that optic nerve exposure and direct physical contact are needed to induce injury. In the ocular blast model, compressed air is directed against the eye while the mouse is housed in a rigid PVC tubing^15^. This method allows for quantification of the force delivered to the ocular surface and is an indirect form of optic nerve injury since the optic nerve is not exposed or comes into contact with an instrument. The major drawback is that the frontal blast leads to severe anterior and posterior segment ocular injuries, and is associated with high mortality rates (ranging from 24–46% depending on the level of air pressure applied)^15^. While each of these models produces the end result of retinal ganglion cell (RGC) death, they fail to accurately simulate the clinical scenario of indirect closed-head injury to the optic nerve^18^.

An ideal TON injury animal model should fulfill the following criteria: 1) simulates the clinical scenario of indirect closed-head trauma and pathological consequences found in the human condition; 2) quantifiable and tunable *ab external* application of force to induce a focal optic nerve lesion without any collateral ocular injuries or mortality; 3) no surgical exposure or direct contact of the optic nerve to induce injury; 4) simple and reproducible; and 5) objective parameters to quantify and characterize the time course of retinal ganglion cell and axonal loss. The primary purpose of this study was to present a novel indirect closed-head TON animal model using an ultrasonic energy transducer to deliver a quantifiable concussive force against the optic nerve. The design of the nerve injury model was to fulfill the above desirable criteria for an ideal animal model that closely approximates the clinical scenario of TON.

## Results

### Sonication-induced TON (SI-TON) in a mouse model

**Figure 1:**
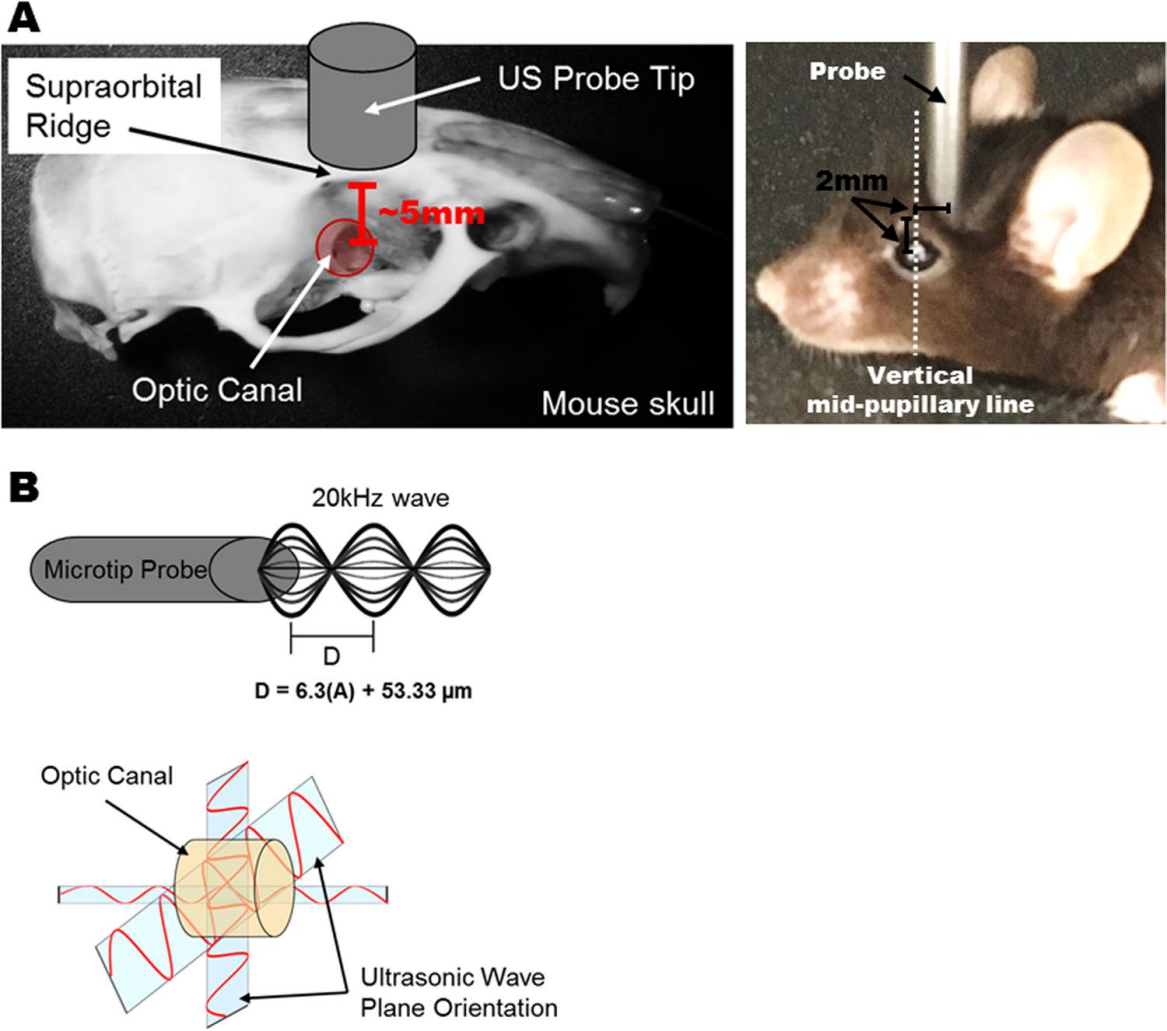
Introducing Traumatic Optic Neuropathy with Sonication. (**A**) To trigger traumatic optic neuropathy (TON), a microtip probe (3 mm) of a laboratory sonifier was placed on the supraorbital ridge (black arrow), directly above the optic nerve’s entrance into the optic canal (left panel). The distance between the orbital rim and optic canal is about 5 mm in 3 month-old C57BL/6 animals. A representative picture of the 3 month-old C57BL/6 mice that were anesthetized and induced for TON by ultrasound (right panel) is shown. (**B**) Schematic illustration of the energy of the ultrasound wave generated from the microtip probe. 20 kHz ultrasound waves are delivered through the bone and concentrated at the fixed portion of the optic nerve at the entrance to the optic canal.

To trigger TON, a laboratory sonifier (Branson Digital Sonifier 450, Connecticut, USA) with microtip probe (3 mm diameter) was placed on the supraorbital ridge at 2mm medial, and 2mm caudal to the vertical mid-pupillary line (Fig. 1A) of each isoflurane-anesthetized mouse. Because the supraorbital ridge is the most anatomically accessible point for stimulation of the optic canal, the ultrasound pulse can be delivered directly through the bones of the optic canal and absorbed by the optic nerve. The sound energy is concentrated focally at the entrance of the optic canal (Fig. 1B). No orbital fractures were detected after sonication session. Similarly, no globe injuries were observed at the energy levels evaluated. No mice died as a direct result of the ultrasound injury at the set energy outputs. The sonifier was set at 35% amplitude for 500 ms, resulting in a modelled 230–250 microns of oscillation at either 120 or 160 Watt output, delivering a precisely quantifiable amount of energy of 60 or 80 Joules, respectively to the injury site with each pulse.

**Figure 2:**
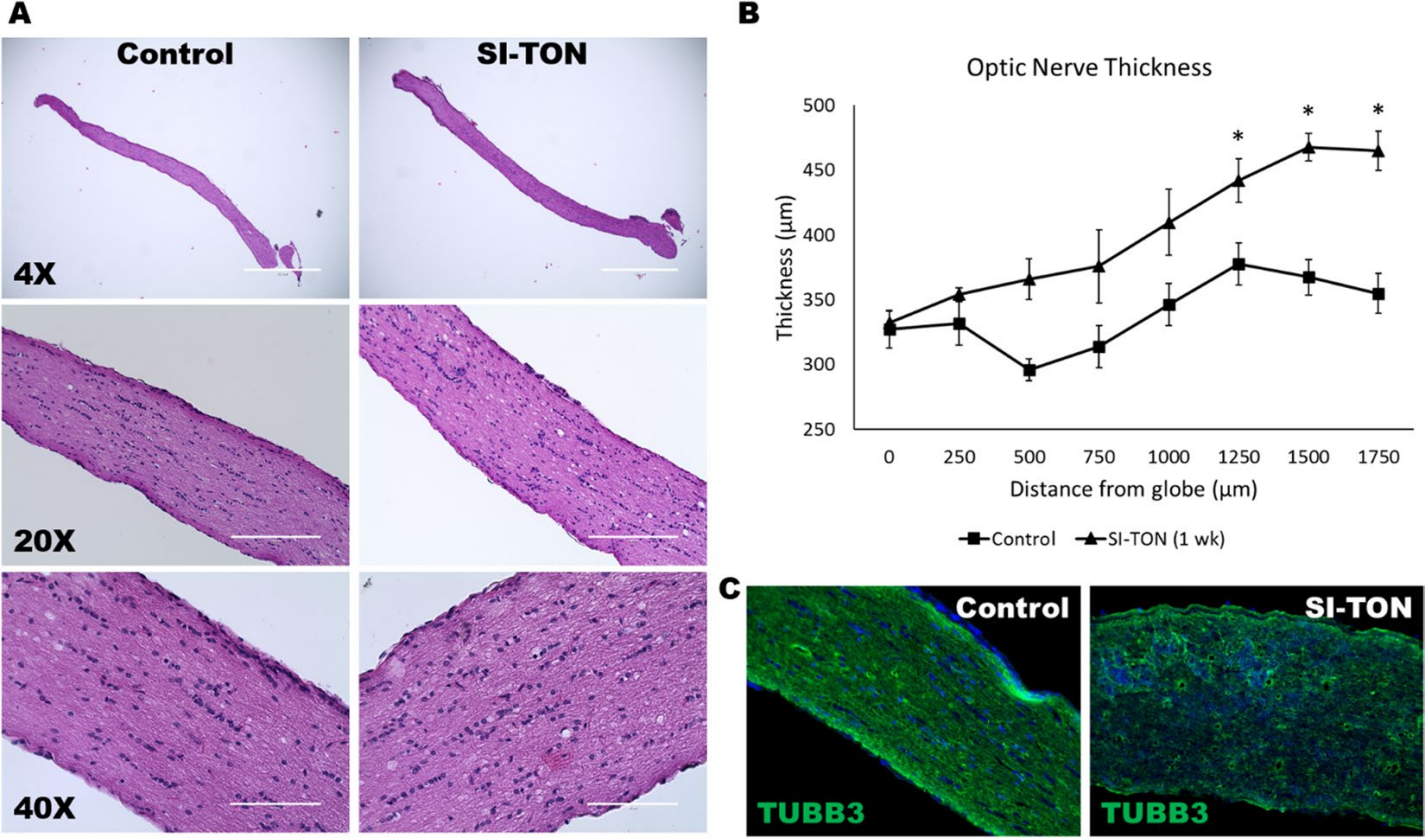
Gross Morphological Changes in the Optic Nerve After SI-TON. (**A**) Gross histological images of normal (control), and SI-TON nerves at 1 week post-injury. (**B**) Morphometric measurements of optic nerve thickness in control and SI-TON nerves (1 week post-injury), showing increasing nerve thickness distally from the globe towards the optic canal (with statistically significant thickening of the nerves past 1mm away from the globe). (**C**) Class III Beta-tubulin immunohistochemical images of control and SI-TON (1 week post-injury) nerves showing axonal immunoreactivity remains, but axonal projections become disorganized and tortuous following SI-TON. (Values are shown as percentages ± SEM,*p value < 0.05).

Gross histological examination showed morphological changes in the optic nerve one week after SI-TON. By light microscopy of H&E sections, optic nerves in the SI-TON cohort experience swelling and presented with numerous vacuolated/cystic lesions throughout the nerve sections (Fig. 2A). Optic nerve thickness was measured every 250 µm distally from its insertion point on the globe. Nerve thickness was shown to increase in the SI-TON animals distally from the globe (Fig. 2B). Past 1 mm from the globe, the SI-TON nerve thickness was greater than the nerve thickness in control animals (average difference in thickness past the 1 mm mark was + 91.74 ± 13.98 µm in the SI-TON group compared to uninjured controls, p-value < 0.05). Immunohistochemical (IHC) imaging for class III β-tubulin (TUJ1) was performed to trace axonal projections in the optic nerve following SI-TON. This analysis showed that, while high TUJ1 immunoreactivity remains following SI-TON, axonal projections in these nerves become disorganized and tortuous, when compared to the smooth and parallel axonal projections in the control eye (Fig. 2C). The morphological changes in the optic nerve after SI-TON indicated that the swelling of optic nerves occurred near the injury site at the optic canal, and gross histology and immunohistochemistry further confirm the disruption in the ultrastructure of the tissues and a dysfunctional presentation of axonal projections at 1 week post-SI-TON.

### RGC loss in the retina in the SI-TON model over time

**Figure 3:**
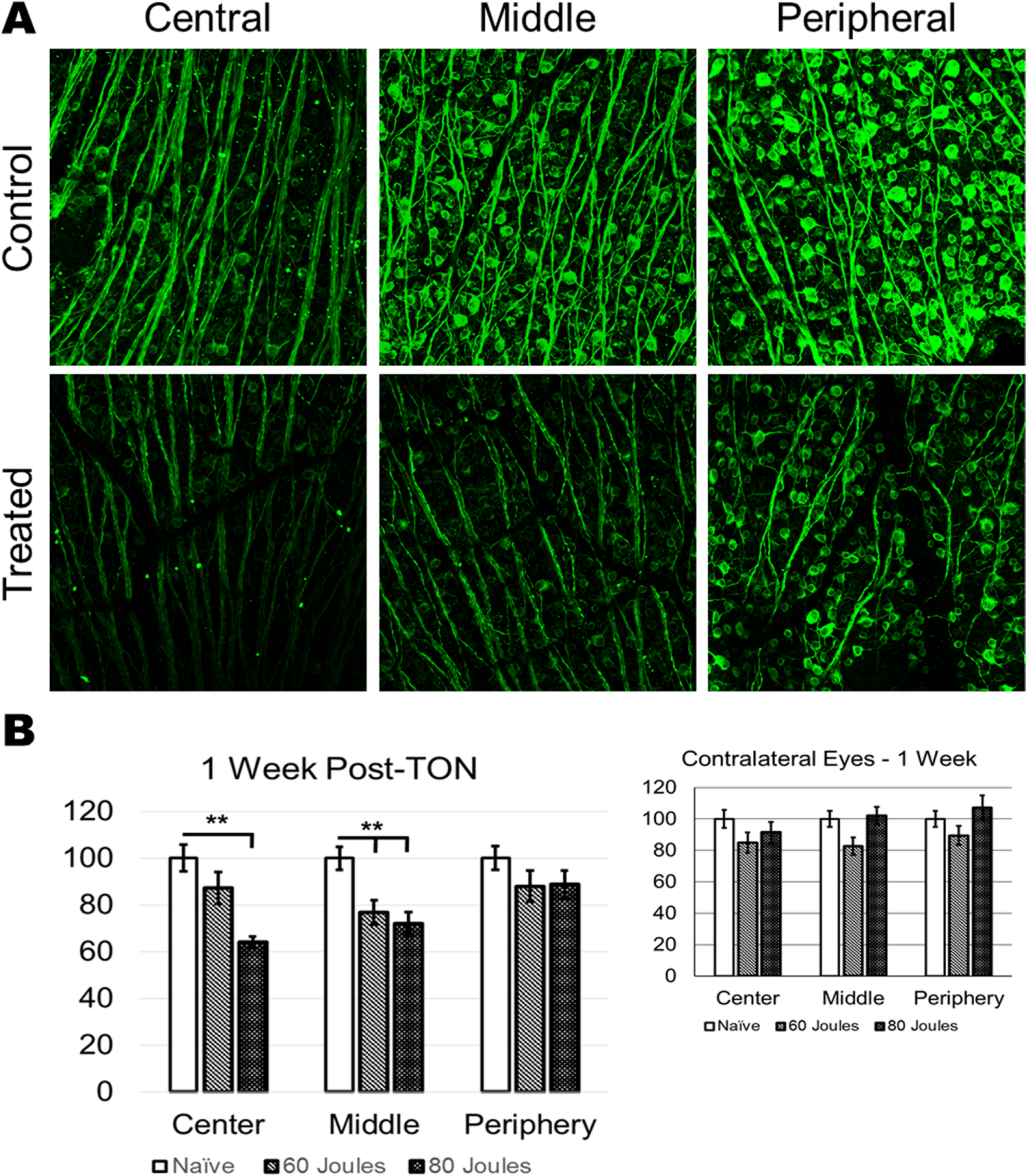
After SI-TON, the number of RGCs in the retina is decreased. (**A**) Representative confocal images of RGCs in the central, middle, and peripheral regions of retinas harvested from treated and control (contralateral) eyes one week after SI-TON injury. RGCs were stained with class III beta- tubulin (TUJ1). (**B**) The number of RGCs in the central, middle, and peripheral retinas of naïve and treated eyes (left panel) and contralateral eyes (right panel) were counted and compared. The graph illustrates the percentage of RGC loss after SI-TON injury compared to naïve (values are shown as percentages ± SEM, *p value < 0.05, **p value < 0.01).

**Figure 4:**
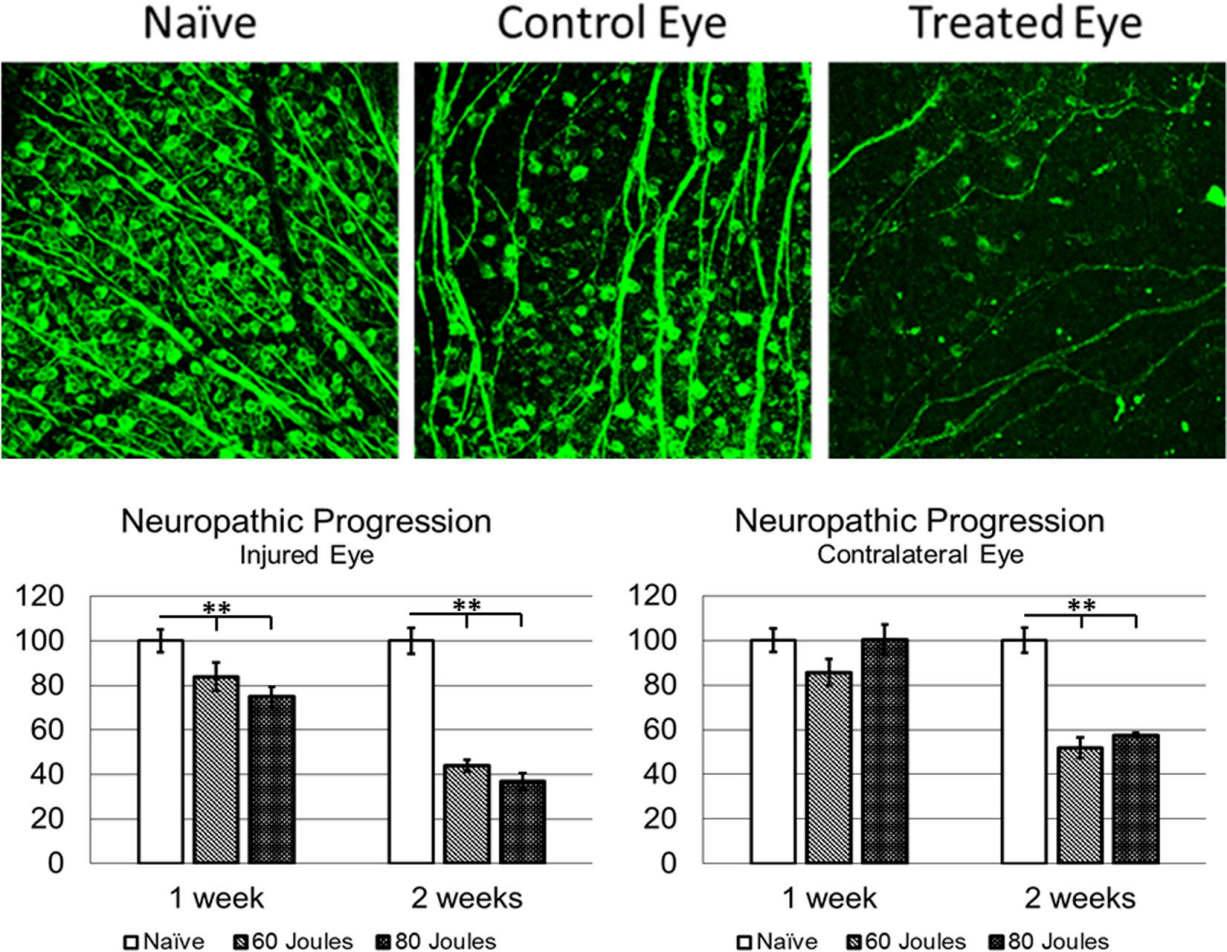
Neuropathic progression of RGC loss in the contralateral eye. (**A**) Representative confocal images of retinas harvested from naïve, control, and treated eyes two weeks after SI-TON injury are shown. Number of RGCs decreased substantially in the treated eyes. RGCs were stained with class III beta-tubulin (TUJ1). Two weeks after SI-TON, numbers of RGCs in the central, middle, and peripheral retina from the naïve (untreated) and treated (with 60- and 80-Joule pulses) eyes (**B**) and contralateral eyes (**C**) were counted and compared. The progressive RGC loss in the contralateral eyes was noted only at 2 weeks after injury. The graph illustrates the percentage of RGC loss after SI-TON compared to naïve (values are shown as percentages ± SEM, *p value < 0.05, **p value < 0.01).

Since traumatic optic neuropathy leads to death of retinal ganglion cells (RGCs), we evaluated the effects of the SI-TON on the RGC survival. RGC staining for class III beta-tubulin (TUJ1) was performed through immunohistochemistry study of retinal flat mounts, and quantified for RGC loss using image analysis software (Image J^16^). One week following SI-TON, the number of RGCs was found to be significantly decreased in the central and middle regions of the retina in the treated eye of the injured cohort, with RGC counts of 87 ± 6% (p-value = 0.345) and 76 ± 5% (p-value = 0.006) respectively, with respect to naïve controls in the 60 Joule cohort, and 63 ± 2% (p-value = 0.006) and 72 ± 5% (p-value = 0.001) respectively, in the 80 Joule (J) group. Meanwhile, no significant loss of RGCs was seen in the contralateral eye one week after injury, with the lowest RGC count found in the middle region of the retina of the 60 J group (82 ± 5%, p-value = 0.094) (Fig. 3A,B). Furthermore, progressive RGC loss over time post-injury was assessed. RGCs loss steadily decreased during the 2-week period after SI-TON in the treated eyes, reaching counts relative to naïve controls of 43 ± 3% (p-value < 0.01) in the 60 J group, and 36 ± 4% (p-value < 0.01) in the 80 J animals (Fig. 4A,B). Interestingly, a loss of RGC viability in the contralateral eye two weeks after injury was observed, with RGC counts of 52 ± 5% (p-value < 0.01) and 57 ± 1% (p-value < 0.01) in the 60 J and 80 J groups respectively at 2 weeks post-SI-TON (Fig. 4C). These data indicated that the SI-TON model induces neuropathic progression of RGC loss in the treated eye as well as in the contralateral eye over time.

**Figure 5:**
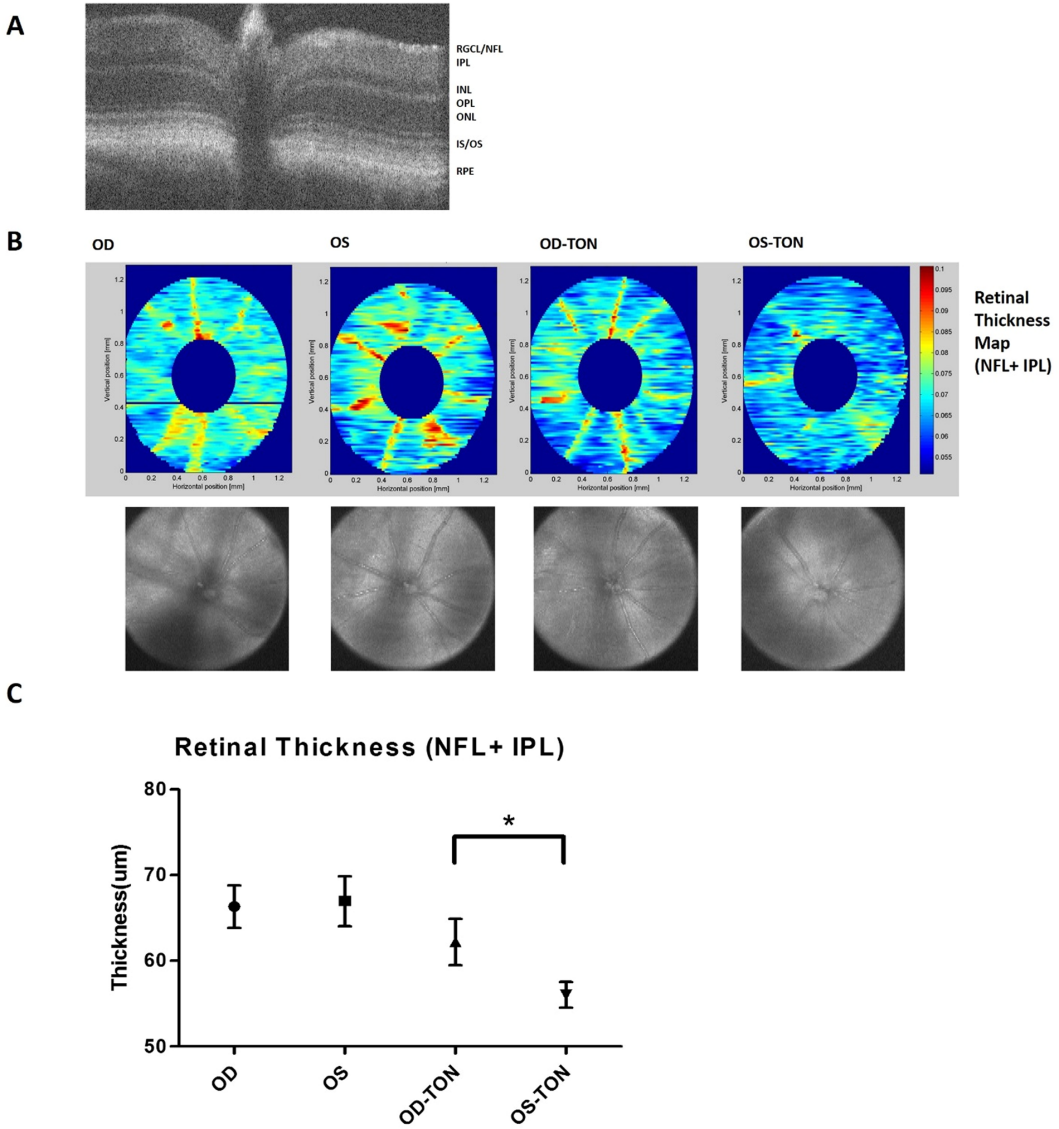
SD-OCT segmentation of retinal layer thinness after SI-TON. (**A**) Representative image of a SD-OCT B-scan retinal cross-section: Retinal layers are labeled as follows: retinal ganglion cell layer/nerve fiber layer (RGCL/NFL), inner plexiform layer (IPL), inner nuclear layer (INL), outer plexiform layer (OPL), outer nuclear layer (ONL), photoreceptor inner segment/outer segment (IS/OS), and Retinal pigment epithelium (RPE). (**B**) Representative retinal thickness heat maps generated from segmentation of the NFL and IP from the right eyes(OD) and left eyes(OS) of normal controls (N = 5) and from the right eyes(OD) and left eyes(OS) of mice treated with ultrasound in the left eye to trigger sonication-induced traumatic optic neuropathy (SI-TON) (N = 5). Fundus images generated by SD-OCT are shown below. (**C**) Retinal thicknesses and standard deviations were plotted with the data from the retinal thickness heat maps. After the SI-TON, the NFL + IPL layers of retinas from treated eye (OS) are significantly thinner than the contralateral controls eye (OD).

In order to further characterize RGC loss, retinal layer thickness were measured one month after SI-TON by SD-OCT as previously described^20^. From earlier study, the RGC histological cell counts correlated with the thickness of combined nerve fiber layer (NFL) and inner plexiform layer (IPL) (Figure 5A)^21^. After SD-OCT measurement, images were segmented manually and the thickness maps for combined NFL and IPL were generated (Fig. 5B). The thickness of combined NFL and IPL was significantly thinner (p-value = 0.04) after SI-TON (56.02 um) compared with controls (62.19 um) (Fig. 5C). Decreased thickness of combined NFL and IPL layers indicated loss of RGC in the retina after SI-TON.

### RGC function is decreased in SI-TON

**Figure 6:**
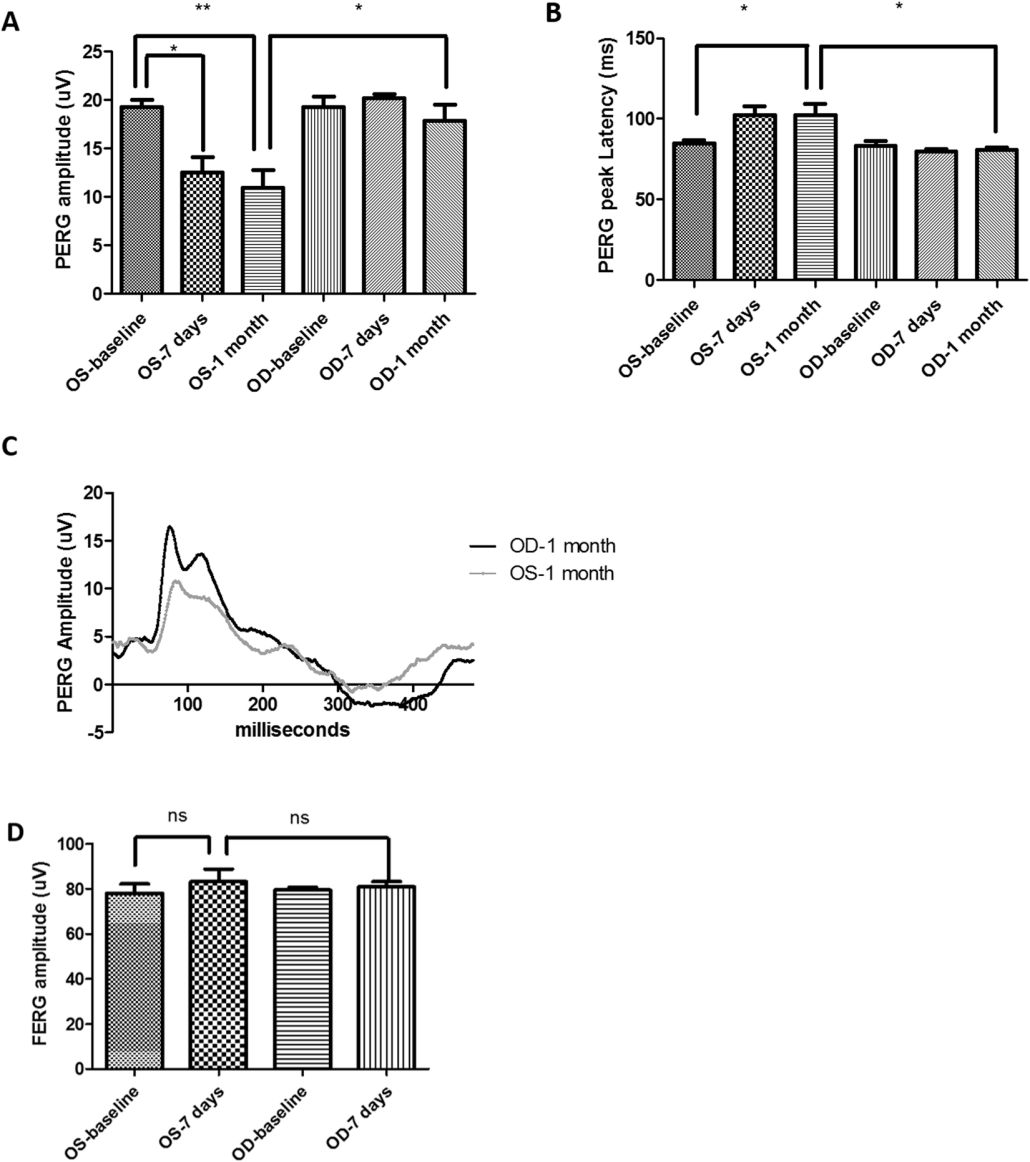
RGC function decreases after SI-TON. Pattern electroretinograms (PERG) were recorded at baseline, 7 days and one month post-TON. The PERG amplitudes (**A**) and PERG peak latency (**B**) at baseline, 7 days and one month post-TON were compared in treated (OS) and contralateral eye (OD) eyes. Representative PERG results were plotted for the treated eye and control eye one month post-TON (**C**). The flash ERG (FERG) at baseline, 7 days post-TON were compared in treated (OS) and contralateral eye (OD) eyes (**D**) (values are shown as Mean ± SEM,*p value < 0.05,* *p value < 0.01, ns: not significant).

To study whether SI-TON affects the function of RGCs, pattern ERG (PERG) recordings were obtained at baseline, one week and one month post-injury. PERG amplitudes were found to continuously decrease over time and were significantly lower in the treated eyes one week after injury, with an additional decrease after one month post-trauma with PERG amplitudes reaching 10.954 ± 2.23 µV in injured eyes, compared to 19.35 ± 0.72 µV at baseline (p-value < 0.01) and 20.23 ± 0.32 µV in the contralateral eyes (p-value < 0.01) (Fig. 6A). A statistically significant increase in the PERG peak latency of injured eyes was similarly observed (Fig. 6B). Representative PERG results were plotted for the injured eye and control eye 1 month post-SI-TON. For the injured eye, the peak is blunter and the waveform was shifted to the right compared with control eye (Fig. 6C). These data suggests that sonication injury triggers a consistent loss of function in the RGC layer. Meanwhile, the flash ERG (FERG) did not indicate any significant change after injury (Fig. 6D), indicating that the function of outer retinal layers remained unaffected by our sonication injury technique. These observations led us to further investigate the molecular events triggered in the nerves by primary injuries, focusing on soluble factors and stress-response cascades in the injured optic nerve.

### SI-TON model induces pro-inflammatory response in the optic nerve

**Figure 7:**
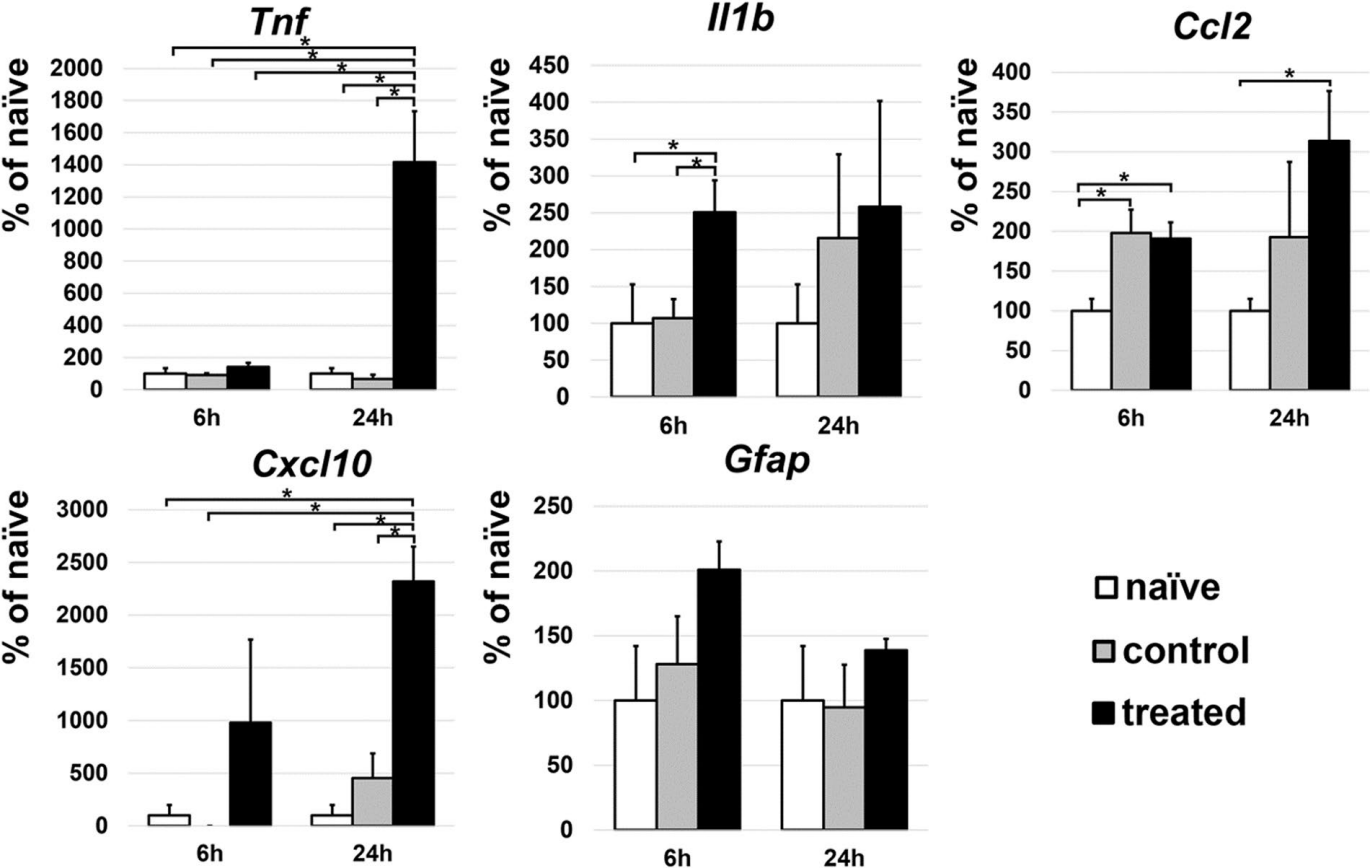
Expression of pro-inflammatory cytokines in the optic nerve. Gene expression profiling of *Tnf*, *Il1b*, *Ccl2*, *Cxcl10*, and *GFAP* at 6 and 24 hr post SI-TON. For each assayed gene, results are presented as percentage expression compared to naive ± SEM of the corresponding values (*p-value < 0.05).

**Figure 8:**
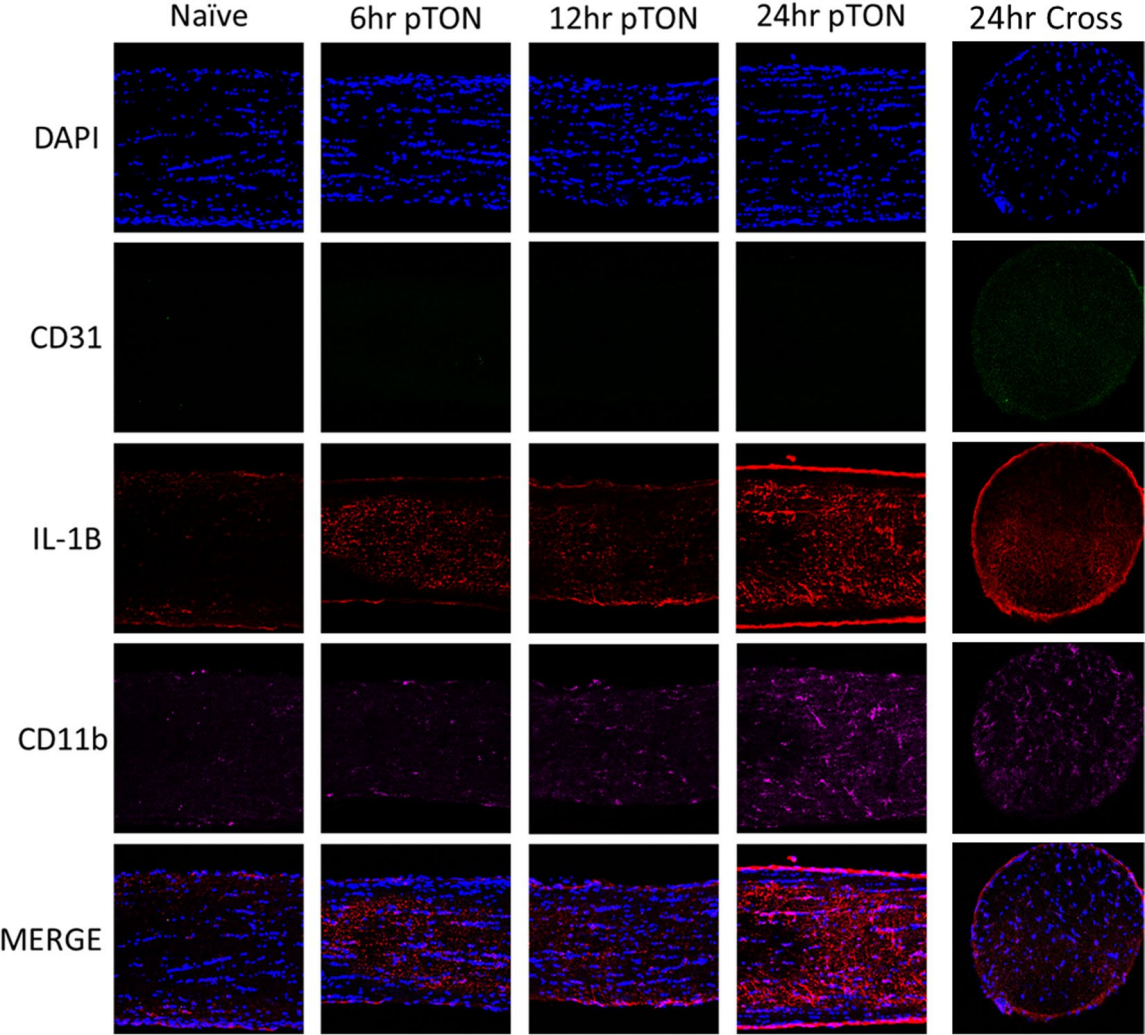
Activation of an inflammatory response in the optic nerve after SI-TON. Confocal images of IHC staining of longitudinal (1–4 columns) as well as transverse (5th column) optic nerve sections from control and treated eyes 1 week after SI-TON.

**Figure 9:**
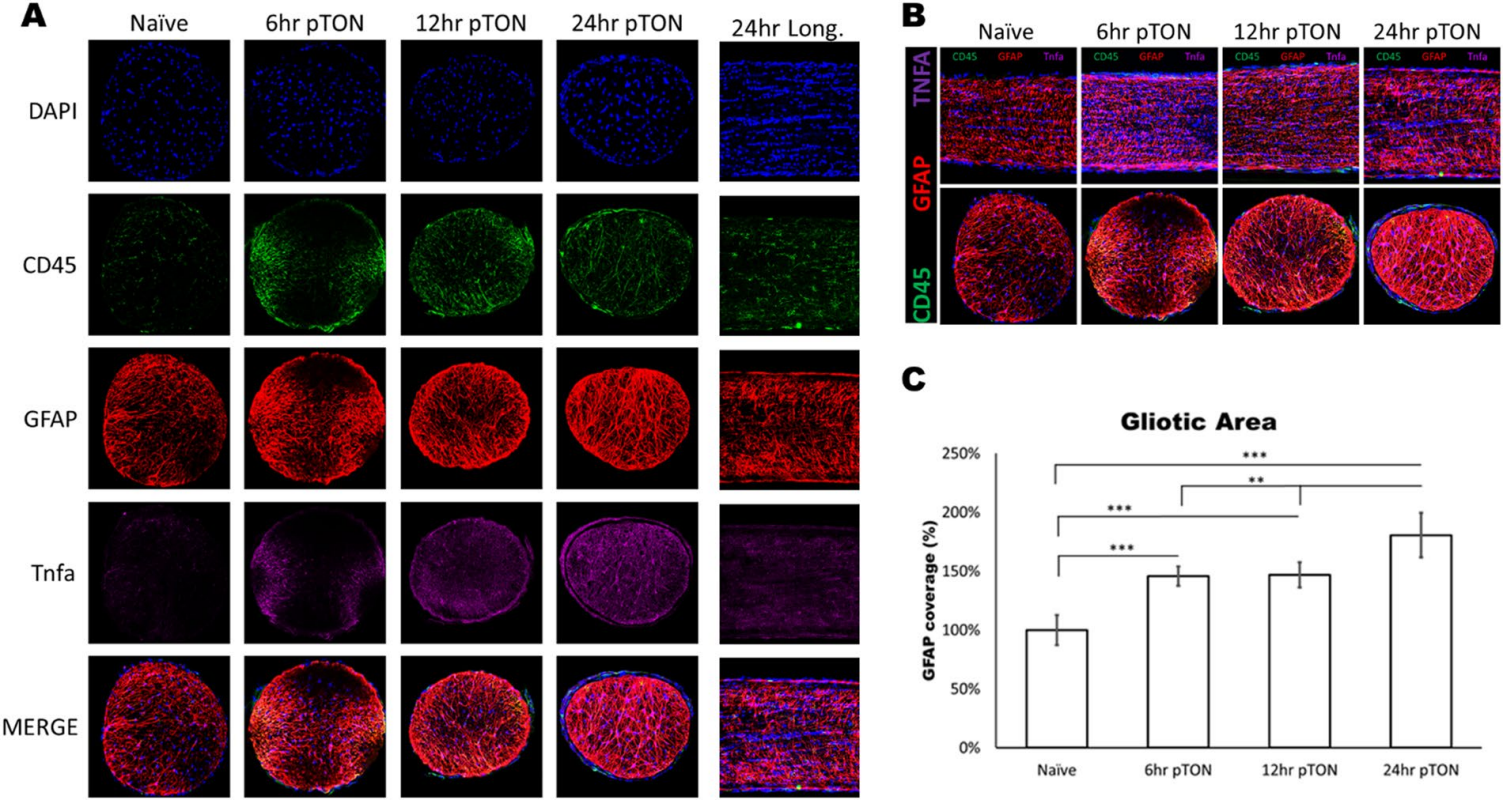
Optic nerve developed a gliotic area after SI-TON. (**A**) Confocal images of IHC staining of longitudinal and transverse optic nerve sections at different time points (naïve, 6, 12, and 24 hours post-TON) are shown. Infiltrating macrophage marker CD45 (green, 2nd row), astrocyte marker GFAP (red, 3rd row), and inflammation marker TNF-alpha (Tnf, purple, 4^th^ row) were stained in each optic nerve section. DAPI were used to counterstain the nuclei. All channels are merged into combined pictures (4^th^ row). (**B**) Transverse (Top row) and longitudinal (Bottom Row) merged images of IHC staining for TNF-alpha (purple), GFAP (red), and CD45 (green) at different time points (naïve, 6, 12, and 24 hours post-TON) are shown. (**C**) The GPAF positive gliotic area in the optic nerve after SI-TON was then measured and quantified. (Values are shown as percentages ± SEM,*p value < 0.05, **p value < 0.01, ***p value < 0.0001).

To characterize the early pathophysiological mechanisms leading to RGC loss and visual function deficit in the SI-TON model, quantitative RT-PCR (qRT-PCR) analysis was performed at 6 and 24hr following injury on selected markers. QRT-PCR analysis showed an upregulation of several pro-inflammatory moieties in the injured nerve (Fig. 7). Interleukin 1-beta (*Il1b*) was significantly upregulated at the 6 hr time point in SI-TON nerves as compared to the uninjured contralateral nerve, or naïve controls (p-value = 0.021, and 0.048 respectively). While still increased in expression at the 24 hr time point, this difference was found to be not significant. Chemokine (C-C motif) ligand 2 (*Ccl2*) was found to be significantly upregulated in both nerves of SI-TON animals at the 6 hr time point when compared to naïve uninjured controls (p-value = 0.011 for SI-TON nerve, and 0.022 for contralateral side). This upregulation of *Ccl2* was still statistically significant only in the SI-TON injured nerve after 24 hours post SI-TON (p-value = 0.012). At the 6 hr post SI-TON time point, both chemokine (C-X-C motif) ligand 10 (*Cxcl10*), and tumor necrosis factor-alpha (*Tnf*) were increased in gene expression, though not statistically significant. However, at 24 hr post SI-TON, both *Cxcl10* and *Tnf* were significantly increased in expression within the SI-TON nerves when compared to every other group (p-value < 0.05). *Tnf* at the 24 hr post SI-TON was the most upregulated gene out the markers chosen for profiling (reaching 1550 ± 346% compared to naïve uninjured control, p-value = 0.025). The gene expression profiles for Il1b and Tnf were consistent with the corresponding protein accumulation levels detected by immunohistochemistry (Figs 8 and 9).

To further study the pathophysiology of TON, immunohistochemistry (IHC) was used to stain the optic nerves for several markers including activated microglia marker CD11b, leukocyte common antigen CD45, platelet endothelial cell adhesion molecule (CD31), tumor necrosis factor alpha (Tnf), and the astrocyte marker GFAP (Figs 8 and 9). IHC staining indicated activation of microglia (CD11b) as well as infiltration of CD45-positive leukocytes in the optic nerve after SI-TON within the first 12 hours of neuropathic progression (Figs 8 and 9A). Soluble Tnf protein accumulation was also observed starting at 6 hours post SI-TON and progressing through the 24 hour time point in both cross-section and longitudinal sections of injured nerves (Fig. 9A,B). After 12 and 24 hours, a robust staining of the astrocyte marker GFAP was present in both transverse and longitudinal optic nerve sections (compared to controls at 0 hours; Fig. 9,B). The GFAP-positive gliotic area was measured and quantified, and was found to increase by approximately 50% (p-value = 0.0026) after 6 hours. After 24 hours, the gliotic area had expanded by about 75% (p-value = 0.0002, Fig. 9C). Overall, following the initial inflammatory response, a gliotic response began at the injury site and developed progressively into a gliotic scar, presumably contributing to further constriction of local vasculature and axonal bundles, accentuating the effects of the primary traumatic event to the nerve.

**Figure 10:**
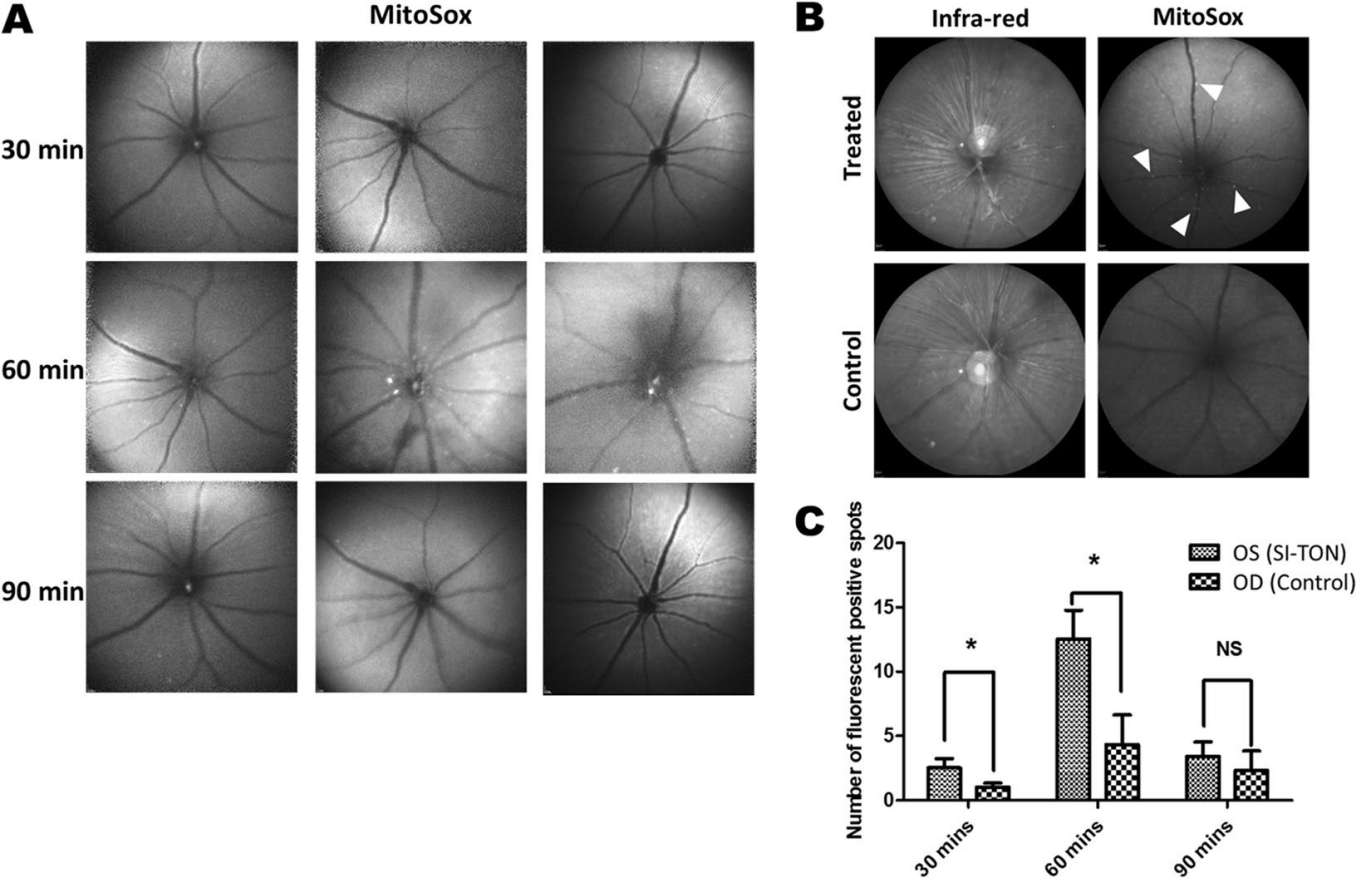
Activation of Reactive Oxygen Species (ROS) in the retina after SI-TON. In eyes injured by the sonication method, ROS were detected as red fluorescence puncta (arrowheads) using Red Mitochondrial Superoxide Indicator (a reactive oxygen species reporter). 1 uL of 200 mM MitoSOX Red Mitochondrial Superoxide Indicator was injected intravitreally into both eyes and images captured by Heidelberg Retinal Tomography (HRT). (**A**) Time course analysis of ROS in the retina after SI-TON. Three representative fluorescence images of ROS are shown for 30, 60 and 90 mins after SI-TON (n = 5). (**B**) ROS imaging at 60 min post SI-TON showing maximal expression of ROS in cells (arrowheads). (**C**) Fluorescent puncta for ROS quantification at 30, 60 and 90 mins after treatment (n = 5). (Values are shown as percentages ± SEM, *p value < 0.05).

Reactive oxygen species (ROS) can rapidly provide indications of a pro-inflammatory stress-response in affected tissues. Using Red Mitochondrial Superoxide Indicator (MitoSox, a dye for ROS), activation of ROS following SI-TON was evaluated using a Heidelberg Spectralis scanning laser ophthalmoscope. ROS activation was detected in the retinas of the treated eyes as early as 30 minutes after injury in SI-TON model (Fig. 10). Such acute upregulation of ROS in the retina may play an important role in RGC death caused by optic nerve injury, and can provide an avenue for immediate therapeutic targeting in early onset of TON, before subsequent neurodegenerative processes supervene. Cytoplasmic accumulation of ROS in RCG or the time course of RGC and axonal loss has not be defined using this model but will be the focus of future experiments using this model.

## Discussion

The development of a new, clinically relevant animal model of indirect traumatic injury to the optic nerve is significant for three reasons. First, achieving the end result of RGC loss in a clinically relevant model allows for the elucidation of the molecular mechanisms that underlie the human condition without the confounding variables of surgical variability and tissue ultrastructure destruction seen in alternative models. Second, the preservation of intact tissue ultrastructure while triggering axonal degeneration and RGC death provides the most permissive environment in which to evaluate new therapeutic strategies to mitigate the effects of trauma and promote RGC survival and axonal regeneration. Third, the tunable level of energies that can be delivered and oriented through the skull makes this model a viable resource for the study of varying degrees of injury manifested over time, involving one or both optic nerves. It might even be adjusted and applied to the study of traumatic brain injury, if so desired.

The *ab external* application of ultrasound energy to the optic nerve in producing RGC loss in mice that simulates the TON clinical scenario, achieves the primary objective of the SI-TON model design. At one week after injury, there were statistically significant declines in RGC numbers in the central and middle retinas of injured eyes when compared to naïve mice that had not been injured with ultrasound. At two weeks after injury, further RGC loss in all areas of the injured retinas was noted. At one week post-injury, there was no significant difference in the RGC counts of the contralateral eyes of treated mice when compared to eyes of treatment-naïve mice was identified. However, at two weeks, a statistically significant decrease in the RGC counts of the contralateral eyes of treatment mice was observed when compared to treatment-naïve eyes. Similarly, a decline in RGC function following injury was observed using pattern electroretinogram (PERG). The PERG output measurement is dominated by the RGC response as has been reported, and functions as a direct measure of RGC electrophysiological function^22^. At one week and one month post-injury, a statistically significant decrease in the a-wave amplitude was noted in injured eyes when compared to the pre-injury, baseline PERG. This observation confirms functional visual impairment following SI-TON injury, correlating with the histopathologic finding of RGC loss. It is worth noting that, while PERG data shows an additional decrease in a-wave amplitude from the 1 week to 1 month time point, this decrease is not as pronounced as the control to 1 week time point decrease, and may indicate a state of resolution of the neurodegenerative process is achieved after several weeks following SI-TON. Immediately after delivering the ultrasonic injury to the optic nerve, soft tissue edema or bleeding on the site of probe contact with skin was observed. Moreover, there were no injury-related deaths using this modality, as compared to other model for recapitulating TON in small animals. Finally, variable intensities of energy employed in this model were used to calculate possible local heating effects on the tissues, as sonication is known to induce rapid heat transfers. Given the reported specific heat capacity for fat, bone, and muscle, the maximum heat increase resulting from a 500 ms, 80 J energy delivery to the supraorbital ridge was calculated to be + Δ1 °C, a negligible temperature difference that would not account for the tissue injuries observed. Because the globes experienced no sequelae from the initial ultrasonic injury, this model should permit better long-term assessment of visual function after trauma directed solely at the optic nerve.

Due to the anatomical proximity of optic nerves in a mouse, one of the drawbacks of this model is the possible scatter of ultrasound energy to the contralateral optic nerve. Consequently, the contralateral optic nerve is a less than ideal control for our animal model – an observation in keeping with other investigators’ recommendation that the contralateral eye should be used with caution as a control in experimental designs studying optic nerve injury^23^. We posit that in a larger animal with a greater distance between optic nerves that there may be less collateral damage from the ultrasonic insult. Furthermore, most commercially available sonifiers have a fixed resonant frequency of the piezoelectric transducer of 20 kHz, which given the disparity between the speed of sound through air (343.2 m/s) and bone (~4,000 m/s), makes the focusing of harmonic waves in such a confined space quite challenging. Ultrasonic transducers capable of delivering variable frequency outputs and intensities can provide further refining over the presented method, but would require expensive custom equipment to achieve. Nevertheless, proper positioning of the mouse with respect to the sonicator microtip is of utmost importance if one is to produce the desired trauma effect to the nerve. In order to reproduce the data generated here, the microtip probe must be positioned properly to align with the entrance of the optic nerve into the canal. Operationally, the probe should be placed over the bony supraorbital ridge approximately 2mm medial, and 2mm caudal to the vertical mid-pupillary line (Fig. 1A). Suboptimal placement of the microtip, either through operator error or movement of the mouse (due to insufficient general anesthesia), can lead to irregular injury by focusing the energy away from the desired target of the optic nerve. Importantly, contact of the probe with the globe during pulse delivery must be completely avoided as this can result in globe rupture and severe co-morbidities that confound optic tract findings. Thus, observed in our model decrease in RGC count on the contralateral nerve may be associated with scatter of energy from the ultrasound delivery, given the close proximity of the mouse optic canals to each other or due to the propagation of soluble pro-inflammatory neurotoxic stimuli throughout the optic nerve tract on both sides over time. Of note, optic nerve crush (ONC) has previously been reported to cause significant RGC loss in the contralateral eye 28 days after injury^23^. ONC would not be expected to cause scatter injury like ultrasound. Rather, it is likely the inflammatory response induced by the optic nerve crush injury that leads to RGC loss on the contralateral side^23^. The statistically significant RGC loss observed in the contralateral eye seen at 2 weeks after SI-TON injury parallels similar observations in ONC mice described by Liu^23^. We postulate that our findings are likely due to the observed post injury inflammatory response hastened by the scatter injury from the ultrasound.

In characterizing this animal model, the aim was to elucidate the cascading sequence of events that occurs after acute injury to the optic nerve. The presence of activated microglia and astrocytes as well as infiltrated leukocytes in optic nerve sections at one week after injury was observed. This finding suggests that the pro-inflammatory cytokines (such as Tnf and Il1b) and chemokines (Ccl2 and Cxcl10) secreted by activated microglia and astrocytes attract pro-inflammatory leukocytes (macrophages) to the injury site. The gradual increases in the expression of the cytokines and chemokines post-injury affirms this damage-triggered inflammatory response. We believe that following the ultrasonic pulse, a secondary sterile inflammatory reaction was elicited at the site of the optic nerve injury. The onset of the sterile inflammatory reaction may be the result of a release of yet-uncharacterized damage associated molecular patterns (DAMPs), activation of toll-like receptor (TLR) response or other pattern recognition receptor (PRR) in the progression of TON neuropathology; a similar mechanism was described previously in the model of retinal ischemia-reperfusion injury^24–28^. We have data showing elevated immunoreactivity for myelin basic protein (MBP) following injury at 1 week post-injury (not shown), which may constitute a possible source of DAMPs. This finding is under evaluation by our laboratory for its capacity to activate such inflammatory cascades in the optic nerve. Similarly, the data presented on infiltrating CD45-positive leukocytes into the injured optic nerve brings into question the compromise of the blood-brain (or blood-retinal) barriers, and the possibility that serum-response may also contribute non-specific DAMPs in triggering this response. This is a rapidly progressing sequence that occurs within the first 24 hours of injury, underscoring the need for early neuroprotective intervention to blunt this molecular response before a deleterious cellular response supervenes in TON patients.

In summary, we have created a novel *ab external* mice injury model for traumatic optic neuropathy (TON) that simulates the mechanism of clinical injuries, confers low mortality and no ocular morbidity when performed correctly, and produces a quantifiable decrease in RGC count. This injury model is simple to implement, and is reproducible. We believe this new model addresses the shortcomings of previous TON animal models and has potential applications in the study of other optic neuropathies and indirect injuries to the head. The characterization of a cascading sequence of inflammatory events following injury and the suggestion of early ROS accumulation in RGC will help better understand the pathophysiology of TON and guide the design of future studies for targeted therapies in neuroprotection.

## Material and Methods

### Animals

All animal experiments were performed in compliance with the NIH Guide for the Care and Use of Laboratory Animals and the ARVO statement for the use of animals in ophthalmic and vision research. Animal protocol was reviewed and approved by the institutional animal care and use committee (IACUC) of the University of Miami. C57BL/6 J mice were obtained from Jackson Laboratory (Bar Harbor, Maine). Mice were housed under ambient conditions (standard humidity and temperature) with a 12 hour light/dark cycle. 3-month-old mice were used for the experiment.

### **I**ntroducing Traumatic optic neuropathy (TON) in a mouse model

Traumatic optic neuropathy (TON) was induced in 3-month-old C57BL/6 J mice with a Branson Digital Sonifier 450 (Branson) by a 3mm microtip probe (Branson) in an acoustic soundproof enclosure chamber (Branson). Before the experiment, the mice were anesthetized by inhaling vaporized isoflurane supplied with oxygen in an induction chamber. The fur adjacent to each mouse’s supraorbital rim was removed with a trimmer. The mice were then placed on the stage of a soundproof enclosure equipped with anesthesia mask. During the procedure, the mice were continually supplied with vaporized isoflurane with oxygen. The microtip probe was placed against each animal’s supraorbital rim above the insertion point of the optic nerve into the optic canal– 2mm medial, and 2mm caudal to the vertical mid-pupillary line. The stage was adjusted and raised so that the microtip probe was in direct contact with the supraorbital ridge, and its weight completely supported by the skull. The sonicator was then activated to deliver a 500msec shock at a 35% or 40% amplitude (resulting in a 230–250 micron oscillation according to manufacturers’ specifications). The contralateral eye was used as a control. Following sonication, the animals were removed from the enclosure and placed in a new cage with thermal support until fully recovered.

### Necropsy and Tissue collection

At given time points (6, 12, 24 hours, and 7 or 14 days after injury, mice were anesthetized by an intraperitoneal injection of ketamine (80 mg/kg of body weight) and xylazine (10 mg/kg of body weight) and perfused through the heart with normal saline. Eyes and optic nerves were then carefully dissected out, taking care not to apply any pressure on the nerves that could affect morphological examination. Optic nerves were dissected in the orbits from their insertion point behind the globe all the way to the optic chiasm, and preserved for immunohistochemistry and quantitative RT-PCR analysis in the appropriate buffers.

### Total RNA isolation and and quantitative RT-PCR

The dissected optic nerves were quickly homogenized in lysis buffer, frozen instantly, and stored in liquid nitrogen until further processing. Total RNA was extracted from optic nerves using the Absolutely RNA Nanoprep kit (Agilent Technologies, Santa Clara, CA, USA), then reverse transcribed with Superscript III polymerase (Invitrogen, USA) to synthesize cDNA. Quantitative RT-PCR was performed in the Rotor-Gene Q Cycler (Qiagen) using SYBR GREEN PCR MasterMix (Qiagen) and gene-specific primers (Table 1). For each gene, relative expression was calculated by comparison with a standard curve, following normalization to the expression of housekeeping gene β-actin (*Actb*; control). We then assessed gene expression 6, and 24 hour’s post SI-TON.

**Table 1:** List of PCR primers.

Gene	Oligonucleotides	
*Il1b*	Forward	GACCTTCCAGGATGAGGACA
	Reverse	AGGCCACAGGTATTTTGTCG
*Tnf*	Forward	CAAAATTCGAGTGACAAGCCTG
	Reverse	GAGATCCATGCCGTTGGC
*Ccl2*	Forward	AGGTCCCTGTCATGCTTCTG
	Reverse	ATTTGGTTCCGATCCAGGTT
*Cxcl10*	Forward	GCTGCAACTGCATCCATATC
	Reverse	CACTGGGTAAAGGGGAGTGA
*Gfap*	Forward	AGAAAGGTTGAATCGCTGGA
	Reverse	CGGCGATAGTCGTTAGCTTC
*Actb*	Forward	CACCCTGTGCTGCTCACC
	Reverse	GCACGATTTCCCTCTCAG

### Immunohistochemistry

Optic nerves were harvested 6, 12, and 24 hours post SI-TON (treated, control, and naïve control) as described previously. Optic nerves were fixed in 4% paraformaldehyde in 1X PBS. After washing in 1X PBS, optic nerves were equilibrated to 10% and then 30% (w/v) sucrose solution and then embedded in OCT medium. Nerves were then frozen, cryosectioned in the longitudinal or cross-sectional planes at a 10 μm thickness, and stored at −80 °C. Sections were thawed at room temperature, washed in 1X PBS, and permeabilized in 0.3% Triton X-100 in PBS for 30 minutes. Tissues were blocked with PBS containing 0.15% Tween 20, 2% bovine serum albumin (BSA), and 5% normal donkey serum for 30 minutes at room temperature. Sections were then incubated with primary antibodies (mouse anti-GFAP, 1:400, Sigma; rat anti-CD45, 1:200, eBioscience; rat anti-CD31, 1:100, BD Biosciences; rabbit anti-IL1 beta, 1:100, Abcam; goat anti-TNFa, 1:50, Santa Cruz; rat anti-CD11b, 1:100, Thermo Fisher Scientific) in blocking solution overnight at 4 °C. After washing in PBS 3 times, sections were incubated with species-specific fluorescent secondary antibodies for 1.5 hours at room temperature. Control sections were incubated with secondary antibody alone. Finally, sections were cover-slipped with Vecta shield (Vector) fluorescent mounting medium containing DAPI. Imaging was performed with a Leica TSL AOBS SP5 confocal microscope (Leica Microsystems).

### Quantification of ganglion cell layer (GCL) neurons in the retina

Whole mount retina were harvested 1 and 2 weeks post-treatment. The orbits were fixed with 4% paraformaldehyde in phosphate-buffered saline (1xPBS) solution for 1 hour and washed with PBS. To make flat mounts, the cornea and crystalline lens were removed, and the entire retina was carefully dissected from the eyecup. Retinas were permeabilized with 0.5% Triton X-100 in 1xPBS for 1 hour then blocked with 0.5% Triton X-100 containing 10% donkey serum in 1xPBS for 1 hour. Whole mount retinas were incubated overnight at 4 °C in a mixture of rabbit monoclonal anti- Beta III Tubulin (TUJ1) antibody (COVANCE, 1:500) reconstituted in 0.2% Triton X-100 in 1xPBS (pH = 7.4) containing 10% donkey serum. After 3 washes with 1xPBS, the retinas were treated with the secondary donkey anti-rabbit antibody for 1.5 hours at room temperature in 1xPBS with 0.15% Tween 20 in the dark. Flat-mounted retinas were washed in 1xPBS with 0.15% Tween 20, then placed on glass slides (with the RGC layer facing up).Four radial cuts were made from the edge to the equator of the retina to make it flat. Retinas were mounted with Fluoromount G and cover slipped. Beta III Tubulin-positive neurons in the ganglion cell layer (GCL) were imaged using a Leica TSL AOBS SP5 confocal microscope (Leica Microsystems, Exton, PA). Individual retinas were sampled randomly to collect a total of 20 images located at the same eccentricity in the four retinal quadrants. Cell loss in the treated eyes’ retinas was calculated as the percentile of the mean cell density in control eyes. ImageJ software was used for the counting of RGC numbers.

### Spectral Domain Optical Coherence Tomography (SD-OCT)

The thicknesses of ganglion cell layers (GCLs) were measure by SD-OCT^20^. Before the experiments, animals were anesthetized by an intraperitoneal injection of ketamine (80 mg/kg of body weight) and xylazine (10 mg/kg of body weight). A 10% phenylephrine solution was then applied to each eye to dilate the pupils. Artificial tears were applied every 2 minutes to keep the corneas moist. Anesthetized mice were mounted with their teeth over a metal rod. Left or right eyes were kept inclined in a 30–45 degree position with respect to the ultrasound gun’s axis. Raster scans were performed for each eye with the following parameters: 512 × 128 (horizontal × vertical) and 1024 × 64 depth scan patterns, with the fast scan in the horizontal direction. After acquisition of the images, mice were kept at 37 °C using a heating pad and monitored until they awoke. High quality images were analyzed and quantified by three-dimensional segmentation software described previously in the literature. Thicknesses of retinal ganglion cell and inner plexiform layers from baseline and 2 weeks after the injury were measured and plotted on a graph.

### Pattern Electroretinogram (PERG)

The electrophysiological function of ganglion cell layers (GCLs) was measured by PERG^22^. Before the experiments, animals were anesthetized by an intraperitoneal injection of ketamine (80 mg/kg of body weight) and xylazine (10 mg/kg of body weight). Mice were restrained by using a bite bar and a nose holder that allowed unobstructed vision. The animals were kept at a constant body temperature of 37 °C with a feedback-controlled heating pad. A drop of balanced salt solution was applied topically as necessary to prevent corneal dryness. PERG signals were recorded from a subcutaneous stainless steel needle placed in the snout. Reference and ground electrodes were inserted under the skin on the back of the head and at the root of the tail, respectively. A visual stimulus of contrast-reversing gratings (covering an area of 56° vertical by 63° horizontal) was aligned approximately on the projection of the optic disk at a viewing distance of 10 cm and was invisible to the contralateral eye. Pattern stimuli were identical for each eye, except for the reversal frequencies (right eye, 0.992 Hz; left eye, 0.984 Hz). Retinal signals were amplified (10,000-fold) and band-pass filtered (1–300 Hz, 6 dB/oct). Three consecutive responses to 600 contrast reversals each were recorded. The pERG responses were superimposed automatically to check for consistency and then averaged. The major positive (P1) and negative waves (N2), the sum of their absolute values (peak-to-trough amplitude), and the peak latency of the major positive wave (P1) were automatically analyzed, calculated, and graphed using MATLAB software (MathWorks).

### Detection of ROS (reactive oxygen species) in the Retina by Heidelberg Retinal Tomography (HRT)

ROS in the retina was detected by fluorescent probes with HRT. TON was triggered in the left eye of the mouse by sonication as described previously. Mice were anesthetized by an intraperitoneal injection of ketamine (80 mg/kg of body weight) and xylazine (10 mg/kg of body weight). Immediately after sonication, 1 ul of 200 mM MitoSOX Red Mitochondrial Superoxide Indicator was injected intravitreally into both eyes. Infrared and Red free (Red Fluorescence) Images were then taken using a Heidelberg Spectralis laser scanning ophthalmoscope (Heidelberg Engineering, Heidelberg, Germany) at 30–40 minutes after the ultrasonic injury.

### Statistical analysis

One-way ANOVA was used for multiple comparisons. The Student t-test was conducted for single comparisons. p-values < 0.05 were considered to be statistically significant.
